# Comparative effectiveness and safety of tenecteplase versus alteplase for intravenous thrombolysis in acute ischemic stroke: a retrospective study

**DOI:** 10.3389/fneur.2025.1691168

**Published:** 2025-10-13

**Authors:** Weili Zhao, Xi Zhang, Yang Zhao, Ruijie Zhao, Yalin Liu

**Affiliations:** Department of Neurology, Xingtai People's Hospital, Xingtai, China

**Keywords:** tenecteplase, alteplase, intravenous thrombolysis, acute ischemic stroke, stroke

## Abstract

**Background:**

Tenecteplase has been proposed as a practical alternative to alteplase for intravenous thrombolysis in acute ischemic stroke. Although randomised trials have demonstrated noninferiority, data from real-world comparative cohorts remain limited.

**Aim:**

This study evaluated functional and safety outcomes of tenecteplase compared with alteplase in a single-center cohort.

**Methods:**

We retrospectively analysed consecutive patients with acute ischemic stroke who underwent intravenous thrombolysis with either tenecteplase or alteplase between April 2023 and April 2025. The primary endpoint was excellent functional recovery at 90 days, defined as a modified Rankin Scale (mRS) score of 0–1. Secondary endpoints included functional independence (mRS 0–2), early neurological improvement, and symptomatic intracranial hemorrhage (sICH). Multivariable logistic regression was used with adjustments for age, baseline NIHSS, diabetes, and atrial fibrillation. Subgroup analyses were performed by age, baseline stroke severity, and history of prior stroke.

**Results:**

A total of 226 patients were included, of whom 147 received alteplase and 79 received tenecteplase. Patients receiving alteplase were older (68 vs. 65) and more frequently had diabetes (49.0% vs. 34.2%) or atrial fibrillation (18.4% vs. 7.6%). At 90 days, good functional outcome (mRS 0–2) was achieved in 27.9% overall (31.6% tenecteplase vs. 26.0% alteplase) and excellent outcome (mRS 0–1) in 20.4% (22.2% vs. 19.3%). Early neurological improvement occurred in 35.8% (42.2% vs. 31.6%). Rates of sICH were low (6.1% vs. 2.5%) and not significantly different after adjustment (aOR 0.41, 95% CI 0.06–1.72). After multivariable adjustment, tenecteplase did not show a statistically significant association with excellent functional recovery (aOR 1.42, 95% CI 0.75–2.70; *p* = 0.280) or with functional independence (aOR 1.57, 0.88–2.83; *p* = 0.131). Tenecteplase was linked with functional independence in patients with severe stroke (aOR 4.12, 95% CI 1.10–17.95; *p* = 0.044).

**Conclusion:**

Tenecteplase demonstrated comparable safety and functional outcomes to alteplase, with signals of potential benefit in patients with more severe strokes. These findings reinforce trial evidence supporting tenecteplase as a practical and effective alternative to alteplase.

## Introduction

Intravenous thrombolysis is a recommended therapy for acute ischaemic stroke within 4.5 h of onset (1–3). Alteplase has been the standard thrombolytic agent for approximately three decades and remains the only drug formally licensed for this indication in most countries. Alteplase acts by binding fibrin and converting plasminogen to plasmin, thereby promoting clot breakdown and vessel recanalisation. However, recent studies have questioned the benefits of alteplase, highlighting modest recanalisation rates (4, 5), the risk of symptomatic intracranial haemorrhage (6). Additionally, alteplase also has practical drawbacks that its short plasma half-life necessitates a bolus followed by a one-hour infusion, which can delay workflow in hyperacute care and poses additional challenges in pre-hospital or resource-limited settings (7).

Tenecteplase, with a faster onset of action and longer half-life (8), allows single-bolus administration while potentially improving clot selectivity. And tenecteplase has proven to be a safe and effective alternative with the growing evidence. Some trials reported that tenecteplase was not inferior to alteplase with a similar safety profile and effectiveness (9–11). And the EXTEND-IA TNK trial found higher rates of early reperfusion in patients with large vessel occlusion (LVO) undergoing thrombectomy with tenecteplase (0.25 mg/kg) (12). Based on these findings, several international guidelines have stated that tenecteplase can be considered as an alternative thrombolytic option for patients with acute ischaemic stroke (13, 14). While alteplase remains the licensed standard in most regions, the clinical landscape is shifting, and tenecteplase is increasingly being considered in both trial and real-world practice.

Several multicentre RCTs in China (9, 15), have suggested the non-inferiority of tenecteplase compared with alteplase in patients with acute ischaemic stroke. These trials provide robust efficacy and safety data, but their settings differ from routine practice. Participants were treated in comprehensive stroke centres under protocolised pathways with structured follow-up. In contrast, regional hospitals frequently care for older patients with greater comorbidity, longer pre-hospital delays, and more variable resources. This study was therefore undertaken to compare the safety and effectiveness of tenecteplase and alteplase in consecutive patients treated in routine care.

## Method

### Study design

We performed a retrospective, single-centre cohort study at Xingtai People’s Hospital of Hebei Province between April 1, 2023, and April 5, 2025. All consecutive patients who received intravenous thrombolysis for acute ischemic stroke during this interval were screened for eligibility.

### Eligibility criteria

Patients were eligible when they had a confirmed diagnosis of acute ischaemic stroke, were aged ≥18 years, received either intravenous tenecteplase or alteplase as their acute management, and had complete discharge data. All patients received intravenous thrombolysis according to international guideline-recommended and weight-based dosing. Tenecteplase was administered as a single intravenous bolus of 0.25 mg/kg, while alteplase was given at a dose of 0.9 mg/kg, with an initial 10% administered as a bolus and the remaining dose infused over one hour.

Patients were not eligible if they had an alternative final diagnosis (e.g., stroke mimic), if they received thrombolysis as part of a clinical trial protocol with undisclosed allocation, or if key baseline or outcome data were missing.

### Data collection

We collected the following baseline data: demographic data (age, sex, weight, and smoking status), clinical baseline [blood pressure, onset-to-needle time (mins), door-to-needle time (mins)], comorbidities history (hypertension, diabetes, atrial fibrillation, dyslipidemia, chronic kidney disease and prior stroke), medication history (anticoagulants and antiplatelet agents) and stroke characteristics.

Stroke characteristics included pre-modified Rankin Scale (mRS) score, National Institutes of Health Stroke Scale (NIHSS) score, large-artery occlusion status, and site of vessel occlusion based on computed tomography angiography (CTA). Occlusion sites were grouped into proximal lesions (internal carotid, MCA-M1, vertebral, or basilar artery) and distal lesions (MCA-M2 to M4 branches, anterior cerebral artery, or posterior cerebral artery). The mRS ranges from 0 (no symptoms) to 6 (death), and the NIHSS ranges from 0 (no deficit) to 42 (most severe deficit). Severe stroke was defined as NIHSS ≥15. All data was recorded by experienced clinicians.

### Outcomes

The primary outcome was defined as achieving an mRS score of 0–1 at 90 days, representing excellent recovery after intravenous tenecteplase. Secondary outcomes included functional independence (mRS 0–2 at 90 days), the occurrence of symptomatic intracranial hemorrhage (sICH) within 36 h, and early neurological improvement (ENI). Symptomatic ICH was defined as a clinical deterioration of ≥4 points on the NIHSS in association with parenchymal hematoma, subarachnoid hemorrhage, or intraventricular hemorrhage. Early neurological improvement was defined as a decline of at least eight points from the initial NIHSS score or the achievement of a score of 0–1 within 72 h of treatment.

### Statistical analyses

Categorical variables were described as frequencies and percentages, and continuous variables as medians and IQRs. Between-group differences were assessed using the Mann–Whitney U test for continuous variables and the χ^2^ test for categorical variables.

Continuous variables were summarised as medians with interquartile ranges (IQR), while categorical variables were described as counts with corresponding percentages. Group comparisons were performed using the Mann–Whitney U test for continuous measures and the χ^2^test for categorical variables.

We first applied univariable anaylsis to examine associations between treatment group and outcomes. Multivariable logistic regression was then performed, adjusting for variables that were considered clinically relevant *a priori* (age, baseline NIHSS score, diabetes, and atrial fibrillation [AF]) and for those that differed significantly in univariable analysis. Subgroup analyses of functional outcomes were conducted according to prespecified categories, including age (<80 years), baseline stroke severity (mild or moderate vs. severe), prior stroke history, and presence of large-vessel occlusion. A sensitivity analysis was undertaken using propensity score–derived inverse probability of treatment weighting (IPTW) to mitigate the influence of residual confounding. Stabilised weights were derived and applied to fit weighted logistic regression.

Crude odds ratios (cOR) and adjusted OR (aOR) with 95% confidence intervals (CI) were reported. As missing data were minimal (<5% for all variables), we performed a complete-case approach without imputation. Given the secondary analysis was considered exploratory, no correction for multiple testing was undertaken. A two-sided *p* < 0.05 was considered statistically significant. All statistical analyses in our study were performed using R (4.4.1, R Foundation for Statistical Computing, Vienna, Austria).

## Result

During the study period, a total of 226 patients were included, of whom 147 received alteplase and 79 received tenecteplase (male, 59.2% versus 55.7%, *p* = 0.715). The median age was 68 years (IQR 64 to 72) in patients with alteplase and 65 years (IQR 62 to 69) in the tenecteplase (*p* = 0.003). Diabetes (49.0% vs. 34.2%, *p* = 0.046) and AF (18.4% vs. 7.6%, *p* = 0.047) were more common among alteplase-treated patients. Over half of patients in both groups presented with moderate stroke severity (57.1% with alteplase vs. 73.4% with tenecteplase), with median baseline NIHSS scores of 11 (IQR 8 to 15.5) and 12 (IQR 8 to14), respectively. More baseline information is shown in Table 1.

**Table 1:** Baseline characteristics and outcomes of patients.

Variable	Alteplase (*n* = 147)	Tenecteplase (*n* = 79)	*p*-value
Age, median (IQR)	68.0 (64.0–72.0)	65.0 (61.5–69.0)	0.003
Sex, *n* (%)			
Female	60 (40.8)	35 (44.3)	0.715
Male	87 (59.2)	44 (55.7)	
Weight, median (IQR), kg	65.0 (60.0–69.5)	63.0 (59.0–70.0)	0.745
Smoking status, *n* (%)	0.585		
Never	52 (35.4)	32 (40.5)	
Former	34 (23.1)	14 (17.7)	
Current	61 (41.5)	33 (41.8)	
Hypertension, *n* (%)	93 (63.3)	61 (77.2)	0.047
Diabetes, *n* (%)	72 (49.0)	27 (34.2)	0.046
Atrial fibrillation, *n* (%)	27 (18.4)	6 (7.6)	0.047
Dyslipidemia, *n* (%)	50 (34.0)	26 (32.9)	0.984
Chronic kidney disease, *n* (%)	18 (12.2)	3 (3.8)	0.065
Prior stroke, *n* (%)	18 (12.2)	16 (20.3)	0.158
Antiplatelet use, *n* (%)	77 (52.4)	44 (55.7)	0.736
Anticoagulant use, *n* (%)	13 (8.8)	9 (11.4)	0.703
NIHSS at baseline, median (IQR)	11.0 (8.0–15.5)	12.0 (8.0–14.0)	0.589
Stroke severity, *n* (%)			0.030
Mild	17 (11.6)	3 (3.8)	
Moderate	84 (57.1)	58 (73.4)	
Severe (NIHSS ≥15)	46 (31.3)	18 (22.8)	
Large vessel occlusion, *n* (%)	54 (36.7)	31 (39.2)	0.821
Occlusion site, *n* (%)	0.851		
Distal	101 (68.7)	56 (70.9)	
Proximal	46 (31.3)	23 (29.1)	
Onset-to-needle time (mins), median (IQR)	147.0 (141.0–152.0)	147.0 (140.5–154.5)	0.527
Door-to-needle time (mins), median (IQR), min	43.0 (39.0–47.0)	43.0 (38.0–46.0)	0.475
Endovascular therapy, *n* (%)	49 (33.3)	22 (27.8)	0.486
Pre-stroke mRS, *n* (%)			1.000
0–2	123 (83.7)	66 (83.5)	
>2	24 (16.3)	13 (16.5)	
Outcomes			
mRS 0–1 at 90 days, *n* (%)	34 (23.1)	23 (29.1)	0.408
mRS 0–2 at 90 days, *n* (%)	75 (51.0)	46 (58.2)	0.370
Symptomatic ICH, *n* (%)	9 (6.1)	2 (2.5)	0.383
Early neurological improvement, *n* (%)	62 (42.2)	25 (31.6)	0.159

**Figure 1:**
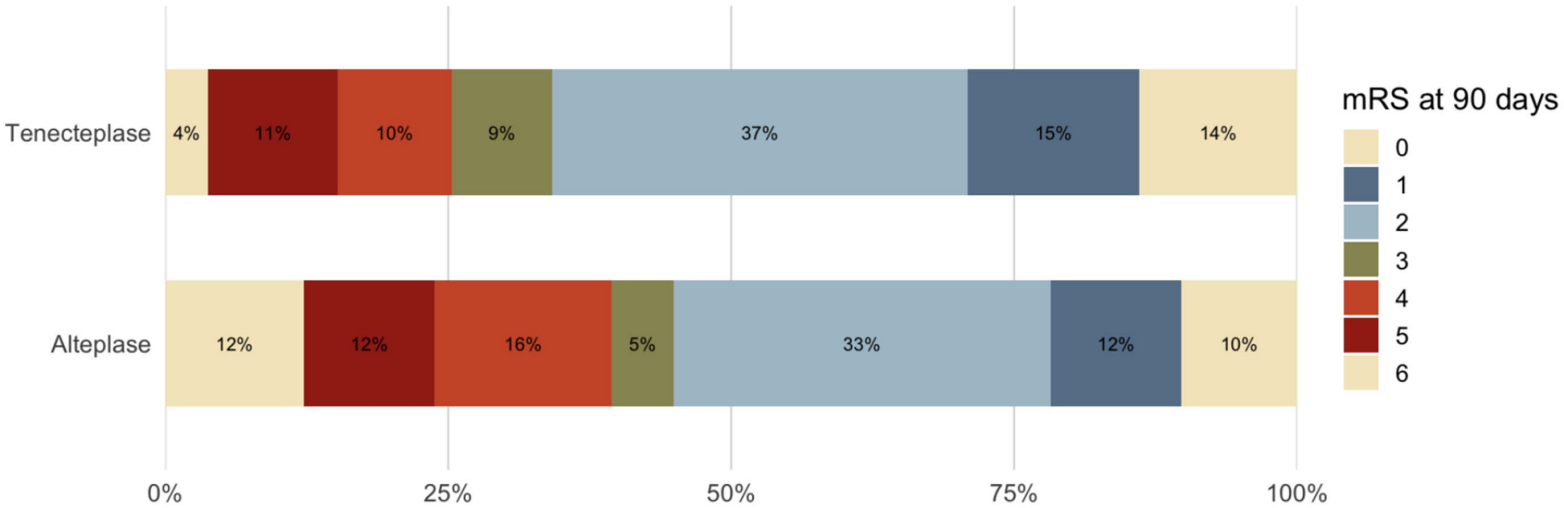
mRS scores in patients with alteplase and with tenecteplase.

At 90 days, 20.4% of patients achieved excellent functional outcome (mRS 0–1), including 22.2% in the tenecteplase treatment group and 19.3% in the alteplase. Good functional outcome (mRS 0–2) was observed in 27.9% of patients overall (31.6% tenecteplase vs. 26.0% alteplase). Early neurological improvement occurred in 35.8% of patients (42.2% vs. 31.6%, respectively). Rates of symptomatic intracranial hemorrhage were low in both groups, at 6.1% in the patients who received tenecteplase group and 2.5% in the alteplase (Figure 1).

In univariable analyses, treatment with tenecteplase was not significantly associated with excellent functional outcome (mRS 0–1) at 90 days (OR, 1.37; 95% CI, 0.73–2.53; *p* = 0.324) or good functional outcome (mRS 0–2: OR, 1.34; 0.77–2.34; *p* = 0.301). Full univariable analyses for all four outcomes are provided in Supplementary Table S1. After adjusting age, NIHSS, diabetes, and atrial fibrillation, no statistically significant positive association was reported for both mRS 0–1 (aOR, 1.42; 0.75–2.70; *p* = 0.280) and mRS 0–2 (aOR, 1.57; 0.88–2.83; *p* = 0.131). These findings suggest a numerical but statistically uncertain trend toward benefit with Tenecteplase (Table 2).

**Table 2:** Unadjusted and adjusted clinical outcomes in patients treated with tenecteplase compared with alteplase.

Outcome	Crude OR (95% CI)	*p*-value	Adjusted OR (95% CI)*	*p*-value
Excellent functional outcome (mRS 0–1 at 90 days)	1.37 (0.73–2.53)	0.324	1.42 (0.75–2.70)	0.280
Good functional outcome (mRS 0–2)	1.34 (0.77–2.34)	0.301	1.57 (0.88–2.83)	0.131
Early neurological improvement	0.64 (0.35–1.12)	0.122	0.65 (0.35–1.17)	0.153
Symptomatic intracranial hemorrhage	0.40 (0.06–1.59)	0.247	0.41 (0.06–1.72)	0.273

*Adjusted by age, baseline NIHSS score, diabetes, and atrial fibrillation.

Early neurological improvement was observed in 42.2% of patients treated with tenecteplase compared with 31.6% in the alteplase group. In multivariable models, no statistically significant association was observed (aOR, 0.65; 0.35–1.17; *p* = 0.153). Rates of sICH were low and similar between groups (6.1% vs. 2.5%, aOR, 0.41, 0.06–1.72, *p* = 0.273).

After IPTW adjustment (Supplementary Table S2), effect estimates were consistent with the primary regression models. For functional independence (mRS 0–2 at 90 days) and excellent outcome (mRS 0–1), the adjusted model had an aOR of 1.46 (95% CI, 0.78–2.74; *p* = 0.239) and 1.36 (0.68–2.71; *p* = 0.388), respectively.

In patients younger than 80 years (mRS 0–2: aOR, 1.52; 0.84–2.77; *p* = 0.166) and without stroke history (aOR, 1.60; 0.85–3.06; *p* = 0.152), tenecteplase therapy was associated with a nonsignificant trend toward better functional outcomes at 90 days. Patients with severe stroke had significantly higher odds of achieving functional independence at 90 days compared with those receiving alteplase (mRS 0–2: aOR, 4.12; 1.10–17.95; *p* = 0.044). Although no significant differences were found in excellent functional recovery in the LVO subgroup, the adjusted estimate suggested a higher likelihood of achieving mRS 0–1 with tenecteplase (aOR, 1.23; 0.45–3.34; *p* = 0.681). Subgroup analyses are presented in Table 3.

**Table 3:** Subgroup analyses by age, baseline stroke severity, and prior stroke history.

Subgroup	Outcome	Crude OR (95% CI)	*p*-value	Adjusted OR (95% CI)	*p*-value
Age <80 years	mRS 0–1	1.32 (0.70–2.44)	0.386	1.44 (0.75–2.75)	0.268
	mRS 0–2	1.27 (0.73–2.23)	0.400	1.52 (0.84–2.77)	0.166
Non-severe stroke	mRS 0–1	1.42 (0.69–2.92)	0.340	1.50 (0.70–3.20)	0.298
	mRS 0–2	1.05 (0.56–2.00)	0.874	1.27 (0.64–2.54)	0.493
Severe stroke	mRS 0–1	1.22 (0.33–4.10)	0.748	1.06 (0.25–4.13)	0.937
	mRS 0–2	2.60 (0.86–8.61)	0.101	**4.12 (1.10–17.95)**	0.044
None prior stroke	mRS 0–1	1.16 (0.59–2.27)	0.660	1.27 (0.63–2.52)	0.505
	mRS 0–2	1.36 (0.74–2.51)	0.324	1.60 (0.85–3.06)	0.152
Having prior stroke	mRS 0–1	7.73 (1.06–158.91)	0.078	6.63 (0.61–198.38)	0.165
	mRS 0–2	1.29 (0.33–5.10)	0.716	1.01 (0.16–5.61)	0.995
LVO present	mRS 0–1	1.13 (0.43–2.92)	0.800	1.23 (0.45–3.34)	0.681
	mRS 0–2	0.67 (0.27–1.63)	0.379	0.74 (0.29–1.88)	0.532

Bold values indicate statistically significant results (*p* < 0.05).

## Discussion

In this retrospective comparative analysis, treatment with tenecteplase was associated with an observed trend toward increased likelihood of achieving functional independence at 90 days compared with alteplase. The direction of effect estimates was consistent across multiple outcomes, including excellent and good functional recovery, but statistical significance was not achieved following multivariable adjustment.

Our findings are broadly consistent with contemporary randomized trials and complementary real-world evidence evaluating tenecteplase as an alternative to alteplase. The ORIGINAL trial in China (9), the Canadian AcT trial (16), and the UK ATTEST-2 study (3) all demonstrated noninferiority of tenecteplase for 90-day functional outcomes, while TRACE-2 (15) similarly confirmed noninferiority in Chinese patients not undergoing thrombectomy. In addition, the EXTEND-IA TNK trial (12) showed significantly higher early reperfusion rates in patients with large-vessel occlusion, a mechanistically plausible finding given tenecteplase’s enhanced fibrin specificity and longer plasma half-life, which may support more sustained thrombolytic activity in high-clot-burden states. Observational registry analyses (17–19) have further reported comparable or superior outcomes with tenecteplase relative to alteplase without excess hemorrhage. Evidence from systematic reviews and meta-analyses that pooled data from both randomized and observational cohorts also indicates equivalent efficacy and safety between the two agents, with some analyses suggesting a greater likelihood of early recanalization with Tenecteplase (20–22). Collectively, these converging lines of evidence suggest that the debate is no longer about whether tenecteplase matches alteplase in safety and efficacy, but whether its pharmacological and workflow advantages might justify replacing alteplase as the preferred first-line agent. The consistency between controlled trials and emerging real-world data, reinforced by our study, strengthens the external validity of trial findings and underscores tenecteplase’s readiness for broader adoption in routine clinical practice.

The safety outcomes in our cohort were consistent with previous reports. Rates of sICH were low and did not differ significantly between agents. A non-significant increase in hemorrhage with tenecteplase has been reported in some small observational studies, but large RCTs and meta-analyses have not demonstrated a higher risk compared with alteplase (14, 23). This is biologically plausible given that both agents act through fibrinolysis, though tenecteplase’s greater fibrin specificity theoretically reduces systemic fibrinolysis and might even limit off-target bleeding. Indeed, pooled trial data suggest that extracranial bleeding complications are not increased with tenecteplase, and intracranial hemorrhage rates remain similar or slightly lower in some analyses. It is also notable that sICH risk in our cohort was well below the rates historically, likely reflecting improvements in patient selection, imaging use, and protocolized blood pressure management over the past decade.

It is notable that the apparent benefit of tenecteplase among patients with severe stroke, in whom the odds of achieving functional independence at 90 days were significantly higher compared with those treated with alteplase. This finding is aligned with the trial (12), where patients with LVO had higher rates of reperfusion before thrombectomy when treated with tenecteplase. Its stronger fibrin affinity and longer duration of action may help explain this potential advantage, particularly in cases involving larger thrombi. Although our subgroup was modest in size and confidence intervals were wide, the similarity of our results to trial data supports further evaluation of tenecteplase in patients with severe stroke burden. These findings, if demonstrated in large worldwide datasets, could have great implications for thrombolysis strategies in real-world practice.

Additionally, our study did not show statistically reliable differences in functional recovery between tenecteplase and alteplase among patients with LVO, although point estimates continued to favour tenecteplase. This differs from recent trial and pooled analyses, which have suggested higher rates of vessel recanalization and improved functional outcomes with tenecteplase in this group (10, 24). There are several potential explanations for this discrepancy. First, the absence of complete imaging data in our cohort restricted adjustment for key determinants of recanalization and outcome, including clot location, collateral circulation, and thrombus length. Second, the limited size of the LVO subgroup reduced statistical power and increased the likelihood of a type II error. Third, differences at the population level may also be relevant. Much of the supportive trial evidence has been generated in highly controlled Western cohorts, whereas Asian populations may differ in stroke subtype distribution, vascular characteristics, and the prevalence of comorbidities such as intracranial atherosclerosis. These biological and epidemiological variations could influence the therapeutic response to thrombolysis yet remain insufficiently explored in comparative research.

Beyond efficacy and safety, practical considerations strongly favor tenecteplase. Its single-bolus administration avoids the logistical complexity of the bolus-plus-infusion regimen required for alteplase. This reduces the risk of dosing errors, infusion delays, or treatment interruption during interhospital transfer, which are issues particularly relevant in stroke networks where patients frequently require transfer for thrombectomy. In our cohort, door-to-needle times were not prolonged by tenecteplase use, suggesting that it can be integrated smoothly into routine pathways without compromising treatment efficiency. However, our findings also highlight realities often underappreciated in trial settings. Functional outcomes in our patients were less favourable than those reported in recent RCTs, reflecting older age, greater comorbidity, and more variable treatment pathways in provincial hospitals. Despite these differences, the relative treatment effects between tenecteplase and alteplase were consistent with trial findings, with no excess risk of intracranial haemorrhage. This suggests that the efficacy signal observed in RCTs can translate into real-world practice, but also that the absolute benefit of thrombolysis is strongly influenced by patient mix and system-level factors. Expanding access to tenecteplase alone will therefore not be sufficient to close the outcome gap between trial populations and routine practice; parallel efforts to optimise stroke pathways, strengthen pre-hospital triage, and improve vascular risk management remain essential if the full potential of reperfusion therapies is to be realised. By documenting these patterns in an unselected provincial cohort, our study provides complementary evidence that extends the external validity of existing trial data and addresses the current lack of real-world evidence from Asia.

This study has several limitations. First, it was a retrospective, single-centre analysis, which introduces risks of selection bias and limits generalisability. Although we adjusted for key prognostic factors and used inverse probability weighting, residual confounding is likely, as variables such as premorbid functional status, stroke aetiology, and physician preference were not fully captured. Second, the modest sample size restricted statistical power, particularly in subgroup analyses. The apparent benefit of tenecteplase in severe stroke is biologically plausible and consistent with prior trials, but wide confidence intervals mean these results should be viewed as exploratory. Third, imaging data were incomplete, limiting assessment of occlusion site, collateral status, and infarct core, which are established determinants of thrombolysis response and may have influenced subgroup findings. Finally, baseline imbalances in age, diabetes, and atrial fibrillation remained despite adjustment, further raising the possibility of residual confounding. These limitations mean our results should be interpreted cautiously and as complementary to existing trial evidence.

## Conclusion

This study supports the growing evidence that tenecteplase is a viable alternative to alteplase for intravenous thrombolysis in acute ischaemic stroke, offering comparable safety and at least equivalent functional outcomes. While our cohort did not demonstrate statistically significant differences, the consistent direction of benefit and the practical advantages of a single-bolus regimen suggest that tenecteplase may be particularly valuable in real-world clinical pathways where time and simplicity are critical.

## Data Availability

The original contributions presented in the study are included in the article/Supplementary material, further inquiries can be directed to the corresponding author/s.

## References

[1] Campbell BCV Ma H Ringleb PA Parsons MW Churilov L Bendszus M. Extending thrombolysis to 4·5-9 h and wake-up stroke using perfusion imaging: a systematic review and meta-analysis of individual patient data. Lancet. (2019) 394:139–47. doi: 10.1016/S0140-6736(19)31053-0, PMID: 31128925

[2] Emberson J Lees KR Lyden P Blackwell L Albers G Bluhmki E. Effect of treatment delay, age, and stroke severity on the effects of intravenous thrombolysis with alteplase for acute ischaemic stroke: a meta-analysis of individual patient data from randomised trials. Lancet. (2014) 384:1929–35. doi: 10.1016/S0140-6736(14)60584-5, PMID: 25106063 PMC4441266

[3] Muir KW Ford GA Ford I Wardlaw JM McConnachie A Greenlaw N. Tenecteplase versus alteplase for acute stroke within 4·5 h of onset (ATTEST-2): a randomised, parallel group, open-label trial. Lancet Neurol. (2024) 23:1087–96. doi: 10.1016/S1474-4422(24)00377-6 39424558

[4] Tsivgoulis G Katsanos AH Schellinger PD Köhrmann M Varelas P Magoufis G. Successful reperfusion with intravenous thrombolysis preceding mechanical Thrombectomy in large-vessel occlusions. Stroke. (2018) 49:232–5. doi: 10.1161/STROKEAHA.117.019261, PMID: 29212743 PMC5742056

[5] Alves HC Treurniet KM Jansen IGH Yoo AJ Dutra BG Zhang G. Thrombus migration paradox in patients with acute ischemic stroke. Stroke. (2019) 50:3156–63. doi: 10.1161/STROKEAHA.119.026107, PMID: 31597552 PMC6824579

[6] Graham GD. Tissue plasminogen activator for acute ischemic stroke in clinical practice: a meta-analysis of safety data. Stroke. (2003) 34:2847–50. doi: 10.1161/01.STR.0000101752.23813.C3, PMID: 14605319

[7] Zachrison KS Schwamm LH. The promise of tenecteplase in acute stroke: within reach or beyond approval? Med J. (2022) 3:651–5. doi: 10.1016/j.medj.2022.09.005, PMID: 36202099

[8] Tanswell P Modi N Combs D Danays T. Pharmacokinetics and pharmacodynamics of tenecteplase in fibrinolytic therapy of acute myocardial infarction. Clin Pharmacokinet. (2002) 41:1229–45. doi: 10.2165/00003088-200241150-00001, PMID: 12452736

[9] Meng X Li S Dai H Lu G Wang W Che F. Tenecteplase vs alteplase for patients with acute ischemic stroke: the ORIGINAL randomized clinical trial. JAMA. (2024) 332:1437–45. doi: 10.1001/jama.2024.14721, PMID: 39264623 PMC11393753

[10] Bala F Singh N Buck B Ademola A Coutts SB Deschaintre Y. Safety and efficacy of Tenecteplase compared with Alteplase in patients with large vessel occlusion stroke: a Prespecified secondary analysis of the ACT randomized clinical trial. JAMA Neurol. (2023) 80:824–32. doi: 10.1001/jamaneurol.2023.2094, PMID: 37428494 PMC10334294

[11] Warach SJ Ranta A Kim J Song SS Wallace A Beharry J. Symptomatic intracranial hemorrhage with Tenecteplase vs Alteplase in patients with acute ischemic stroke: the comparative effectiveness of routine Tenecteplase vs Alteplase in acute ischemic stroke (CERTAIN) collaboration. JAMA Neurol. (2023) 80:732–8. doi: 10.1001/jamaneurol.2023.1449, PMID: 37252708 PMC10230371

[12] Campbell BCV Mitchell PJ Churilov L Yassi N Kleinig TJ Dowling RJ. Tenecteplase versus Alteplase before Thrombectomy for ischemic stroke. N Engl J Med. (2018) 378:1573–82. doi: 10.1056/NEJMoa1716405, PMID: 29694815

[13] Powers WJ Rabinstein AA Ackerson T Adeoye OM Bambakidis NC Becker K. Guidelines for the early management of patients with acute ischemic stroke: 2019 update to the 2018 guidelines for the early management of acute ischemic stroke: a guideline for healthcare professionals from the American Heart Association/American Stroke Association. Stroke. (2019) 50:e344–418. doi: 10.1161/STR.0000000000000211, PMID: 31662037

[14] Berge E Whiteley W Audebert H De Marchis G Fonseca AC Padiglioni C. European stroke organisation (ESO) guidelines on intravenous thrombolysis for acute ischaemic stroke. Eur Stroke J. (2021) 6:I–LXII. doi: 10.1177/2396987321989865, PMID: 33817340 PMC7995316

[15] Wang Y Li S Pan Y Li H Parsons MW Campbell BCV. Tenecteplase versus alteplase in acute ischaemic cerebrovascular events (TRACE-2): a phase 3, multicentre, open-label, randomised controlled, non-inferiority trial. Lancet. (2023) 401:645–54. doi: 10.1016/S0140-6736(22)02600-9, PMID: 36774935

[16] Menon BK Buck BH Singh N Deschaintre Y Almekhlafi MA Coutts SB. Intravenous tenecteplase compared with alteplase for acute ischaemic stroke in Canada (AcT): a pragmatic, multicentre, open-label, registry-linked, randomised, controlled, non-inferiority trial. Lancet. (2022) 400:161–9. doi: 10.1016/S0140-6736(22)01054-6, PMID: 35779553

[17] Zhong CS Beharry J Salazar D Smith K Withington S Campbell BCV. Routine use of Tenecteplase for thrombolysis in acute ischemic stroke. Stroke. (2021) 52:1087–90. doi: 10.1161/STROKEAHA.120.030859, PMID: 33588597

[18] Psychogios K Palaiodimou L Katsanos AH Magoufis G Safouris A Kargiotis O. Real-world comparative safety and efficacy of tenecteplase versus alteplase in acute ischemic stroke patients with large vessel occlusion. Ther Adv Neurol Disord. (2021) 14:1756286420986727. doi: 10.1177/1756286420986727, PMID: 33488774 PMC7809628

[19] Peng TJ Schwamm LH Fonarow GC Hassan AE Hill M Messé SR. Contemporary Prestroke dual antiplatelet use and symptomatic intracerebral hemorrhage risk after thrombolysis. JAMA Neurol. (2024) 81:722–31. doi: 10.1001/jamaneurol.2024.1312, PMID: 38767894 PMC11106713

[20] Potla N Ganti L. Tenecteplase vs. alteplase for acute ischemic stroke: a systematic review. Int J Emerg Med. (2022) 15:1. doi: 10.1186/s12245-021-00399-w, PMID: 34983359 PMC8903524

[21] Wang L Hao M Wu N Wu S Fisher M Xiong Y. Comprehensive review of Tenecteplase for thrombolysis in acute ischemic stroke. J Am Heart Assoc. (2024) 13:e031692. doi: 10.1161/JAHA.123.031692, PMID: 38686848 PMC11179942

[22] Burgos AM Saver JL. Evidence that tenecteplase is noninferior to alteplase for acute ischemic stroke: meta-analysis of 5 randomized trials. Stroke. (2019) 50:2156–62. doi: 10.1161/STROKEAHA.119.025080, PMID: 31318627

[23] Logallo N Novotny V Assmus J Kvistad CE Alteheld L Rønning OM. Tenecteplase versus alteplase for management of acute ischaemic stroke (NOR-TEST): a phase 3, randomised, open-label, blinded endpoint trial. Lancet Neurology. (2017) 16:781–8. doi: 10.1016/S1474-4422(17)30253-3, PMID: 28780236

[24] Katsanos AH Safouris A Sarraj A Magoufis G Leker RR Khatri P. Intravenous thrombolysis with Tenecteplase in patients with large vessel occlusions: systematic review and meta-analysis. Stroke. (2021) 52:308–12. doi: 10.1161/STROKEAHA.120.030220, PMID: 33272127

