# Supplementary material for: Comparative effectiveness and safety of tenecteplase versus alteplase for intravenous thrombolysis in acute ischemic stroke: a retrospective study
Source: Front Neurol. 2025 Oct 13;16:1691168. doi: 10.3389/fneur.2025.1691168 (PMC12554612; doi:10.3389/fneur.2025.1691168)
Supplement: Supplementary file 1 [file Table_1.DOCX]

Table S1 Univariable analyses of baseline predictors for study outcomes

| **Predictor** | **ENI OR (95% CI)** | **p-value** | **mRS 0–1 OR (95% CI)** | **p-value** | **mRS 0–2 OR (95% CI)** | **p-value** | **sICH OR (95% CI)** | **p-value** |
| --- | --- | --- | --- | --- | --- | --- | --- | --- |
| Age (years) | 0.99 (0.95–1.03) | 0.607 | 1.00 (0.96–1.05) | 0.887 | 1.03 (0.99–1.08) | 0.126 | 0.99 (0.89–1.09) | 0.801 |
| Male sex | 1.22 (0.71–2.11) | 0.477 | 1.10 (0.60–2.04) | 0.766 | 1.15 (0.67–1.95) | 0.615 | 0.59 (0.17–2.01) | 0.394 |
| Hypertension | 0.64 (0.36–1.13) | 0.122 | 1.43 (0.74–2.85) | 0.300 | 0.96 (0.55–1.69) | 0.897 | 2.17 (0.54–14.50) | 0.329 |
| Diabetes | 0.99 (0.58–1.70) | 0.976 | 1.00 (0.54–1.83) | 0.992 | 0.87 (0.51–1.47) | 0.590 | 1.57 (0.46–5.61) | 0.465 |
| Atrial fibrillation | 1.87 (0.89–3.96) | 0.100 | 1.13 (0.47–2.53) | 0.769 | 1.90 (0.89–4.27) | 0.106 | 1.32 (0.20–5.43) | 0.731 |
| Dyslipidemia | 0.83 (0.46–1.46) | 0.514 | 0.88 (0.46–1.66) | 0.705 | 1.30 (0.75–2.29) | 0.351 | 0.73 (0.16–2.61) | 0.649 |
| Prior stroke | 0.53 (0.22–1.15) | 0.122 | 0.59 (0.21–1.43) | 0.274 | 0.97 (0.47–2.04) | 0.939 | 1.27 (0.19–5.22) | 0.766 |
| Baseline NIHSS (per point) | 0.95 (0.91–1.00) | 0.073 | 1.01 (0.95–1.07) | 0.774 | 0.97 (0.92–1.02) | 0.176 | 0.97 (0.86–1.09) | 0.584 |
| Stroke severity – moderate | 0.52 (0.20–1.33) | 0.171 | 1.02 (0.37–3.31) | 0.973 | 0.62 (0.22–1.61) | 0.337 | 0.12 (0.02–0.71) | 0.014 |
| Stroke severity – severe | 0.40 (0.14–1.11) | 0.079 | 1.00 (0.33–3.46) | 1.000 | 0.54 (0.18–1.49) | 0.244 | 0.48 (0.11–2.53) | 0.347 |
| Antiplatelet use | 0.57 (0.33–0.97) | 0.039 | 0.95 (0.52–1.74) | 0.874 | 1.26 (0.75–2.13) | 0.390 | 1.04 (0.31–3.72) | 0.945 |
| Anticoagulant use | 1.12 (0.44–2.72) | 0.807 | 1.81 (0.69–4.48) | 0.210 | 1.05 (0.43–2.58) | 0.921 | 2.17 (0.32–9.16) | 0.343 |
| Large vessel occlusion | 1.20 (0.69–2.08) | 0.520 | 1.56 (0.85–2.88) | 0.151 | 0.71 (0.41–1.22) | 0.215 | 3.07 (0.90–12.04) | 0.081 |
| Proximal occlusion | 1.04 (0.58–1.85) | 0.897 | 1.32 (0.69–2.49) | 0.388 | 0.61 (0.34–1.07) | 0.086 | 2.90 (0.84–10.38) | 0.088 |
| Weight (kg) | 1.01 (0.98–1.05) | 0.448 | 1.00 (0.96–1.04) | 0.940 | 0.98 (0.95–1.02) | 0.345 | 1.01 (0.93–1.09) | 0.858 |
| Smoking – former | 1.61 (0.78–3.32) | 0.201 | 0.79 (0.33–1.82) | 0.587 | 0.92 (0.45–1.87) | 0.818 | 0.69 (0.10–3.33) | 0.661 |
| Smoking – current | 1.18 (0.64–2.18) | 0.602 | 1.15 (0.59–2.26) | 0.688 | 1.47 (0.82–2.68) | 0.201 | 0.70 (0.17–2.74) | 0.608 |
| Pre-stroke mRS >2 | 0.73 (0.34–1.51) | 0.408 | 0.52 (0.19–1.25) | 0.173 | 0.79 (0.39–1.61) | 0.515 | – | – |

Table S2 Subgroup analyses by age, baseline stroke severity, and prior stroke history

| **Subgroup** | **Outcome** | **Crude OR (95% CI)** | **p-value** | **Adjusted OR (95% CI)** | **p-value** |
| --- | --- | --- | --- | --- | --- |
| **Age <80 years** | mRS 0–1 | 1.32 (0.70–2.44) | 0.386 | 1.44 (0.75–2.75) | 0.268 |
|  | mRS 0–2 | 1.27 (0.73–2.23) | 0.400 | 1.52 (0.84–2.77) | 0.166 |
| **Non-severe stroke** | mRS 0–1 | 1.42 (0.69–2.92) | 0.340 | 1.50 (0.70–3.20) | 0.298 |
|  | mRS 0–2 | 1.05 (0.56–2.00) | 0.874 | 1.27 (0.64–2.54) | 0.493 |
| **Severe stroke** | mRS 0–1 | 1.22 (0.33–4.10) | 0.748 | 1.06 (0.25–4.13) | 0.937 |
|  | mRS 0–2 | 2.60 (0.86–8.61) | 0.101 | **4.12 (1.10–17.95)** | 0.044 |
| **None prior stroke** | mRS 0–1 | 1.16 (0.59–2.27) | 0.660 | 1.27 (0.63–2.52) | 0.505 |
|  | mRS 0–2 | 1.36 (0.74–2.51) | 0.324 | 1.60 (0.85–3.06) | 0.152 |
| **Having prior stroke** | mRS 0–1 | 7.73 (1.06–158.91) | 0.078 | 6.63 (0.61–198.38) | 0.165 |
|  | mRS 0–2 | 1.29 (0.33–5.10) | 0.716 | 1.01 (0.16–5.61) | 0.995 |
| **LVO present** | mRS 0–1 | 1.13 (0.43–2.92) | 0.800 | 1.23 (0.45–3.34) | 0.681 |
|  | mRS 0–2 | 0.67 (0.27–1.63) | 0.379 | 0.74 (0.29–1.88) | 0.532 |
